# Supplementary material for: Optimized production and fluorescent labeling of SARS-CoV-2 virus-like particles
Source: Sci Rep. 2022 Aug 27;12:14651. doi: 10.1038/s41598-022-18681-z (PMC9419636; doi:10.1038/s41598-022-18681-z)
Supplement: Supplementary file 1 — Supplementary Information. [file 41598_2022_18681_MOESM1_ESM.docx]

**SUPPLEMENTARY DATA and MATERIALS**

**Optimized production and fluorescent labeling of SARS-CoV-2 virus-like particles**

Manon Gourdelier^1^, Jitendriya Swain^1^, Coline Arone^1^, Anita Mouttou^1^, David Bracquemond^1^, Peggy Merida^1^, Saveez Saffarian^2^, Sébastien Lyonnais ^3^, Cyril Favard^1^ and Delphine Muriaux^1,3^*

*1-Institut de Recherche en Infectiologie de Montpellier (IRIM), Université de Montpellier, CNRS UMR9004, Montpellier, France*

*2- Department of Physics and Astronomy, Center for Cell and Genome Sciences, University of Utah, Salt Lake City, Utah, United States*

*3-CEMIPAI, Université de Montpellier, CNRS UAR3725, Montpellier, France*

*corresponding:delphine.muriaux@irim.cnrs.fr

**
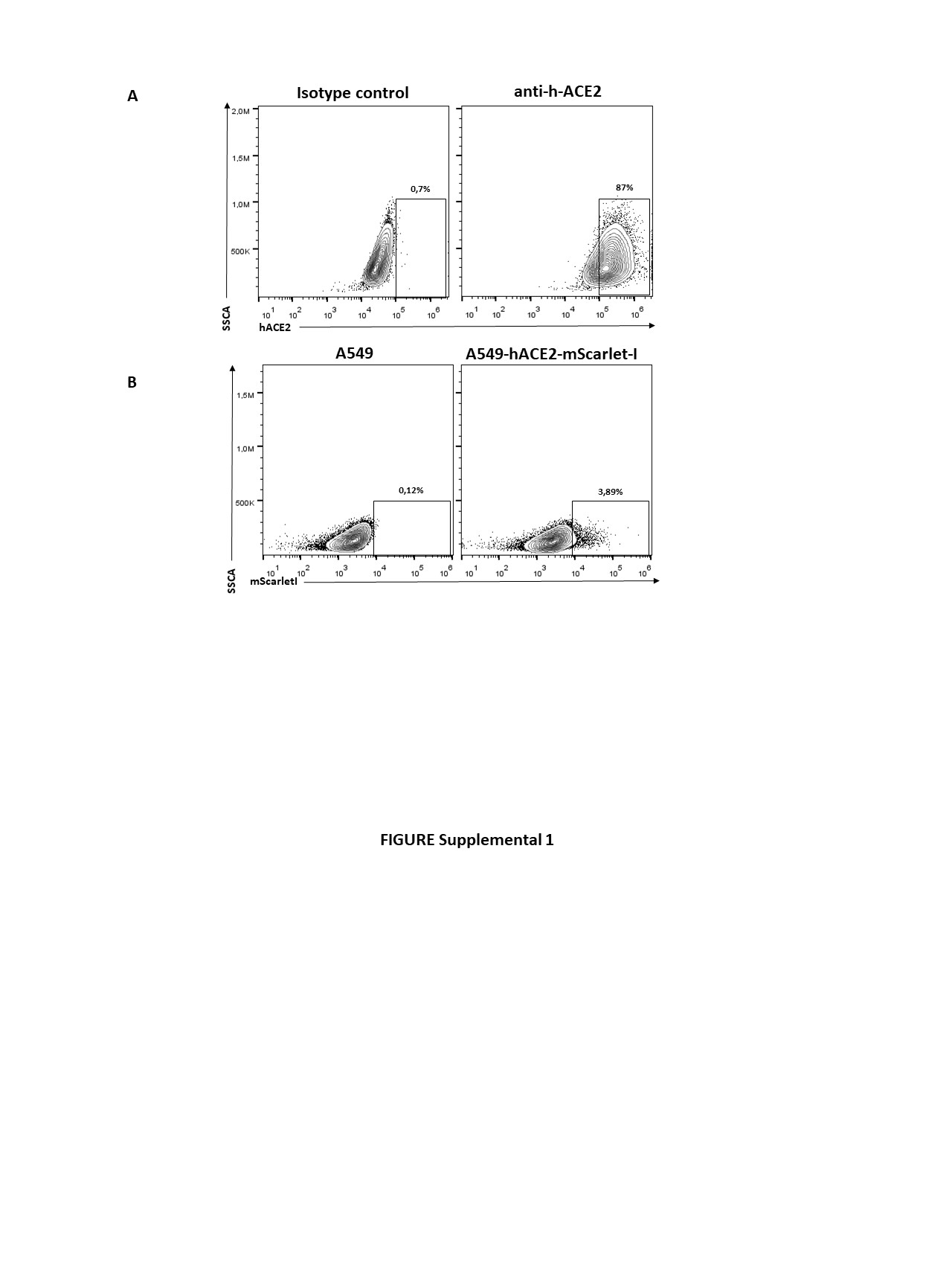
Supplementary Figure 1: Detection of hACE2 and hACE-mScarlet1 at the cell surface of the A549-hACE2 and A549-hACE2mScarlet1 pulmonary cell lines using flow cytometry.** (A) Dot plot representing side scatter (SSC) in function of mean intensity (secondary AF488 antibody). Gate represents the pourcentage of hACE2 positive cells. Cells labeled with only secondary antibody are used as control. (B) Dot plot representing side scatter (SSC) in function of mean intensity of mScarletI (Ext569/Em594nm). Gate represents the pourcentage of mScarletI positive cells. Non transduced A549 cells are used as control.

**
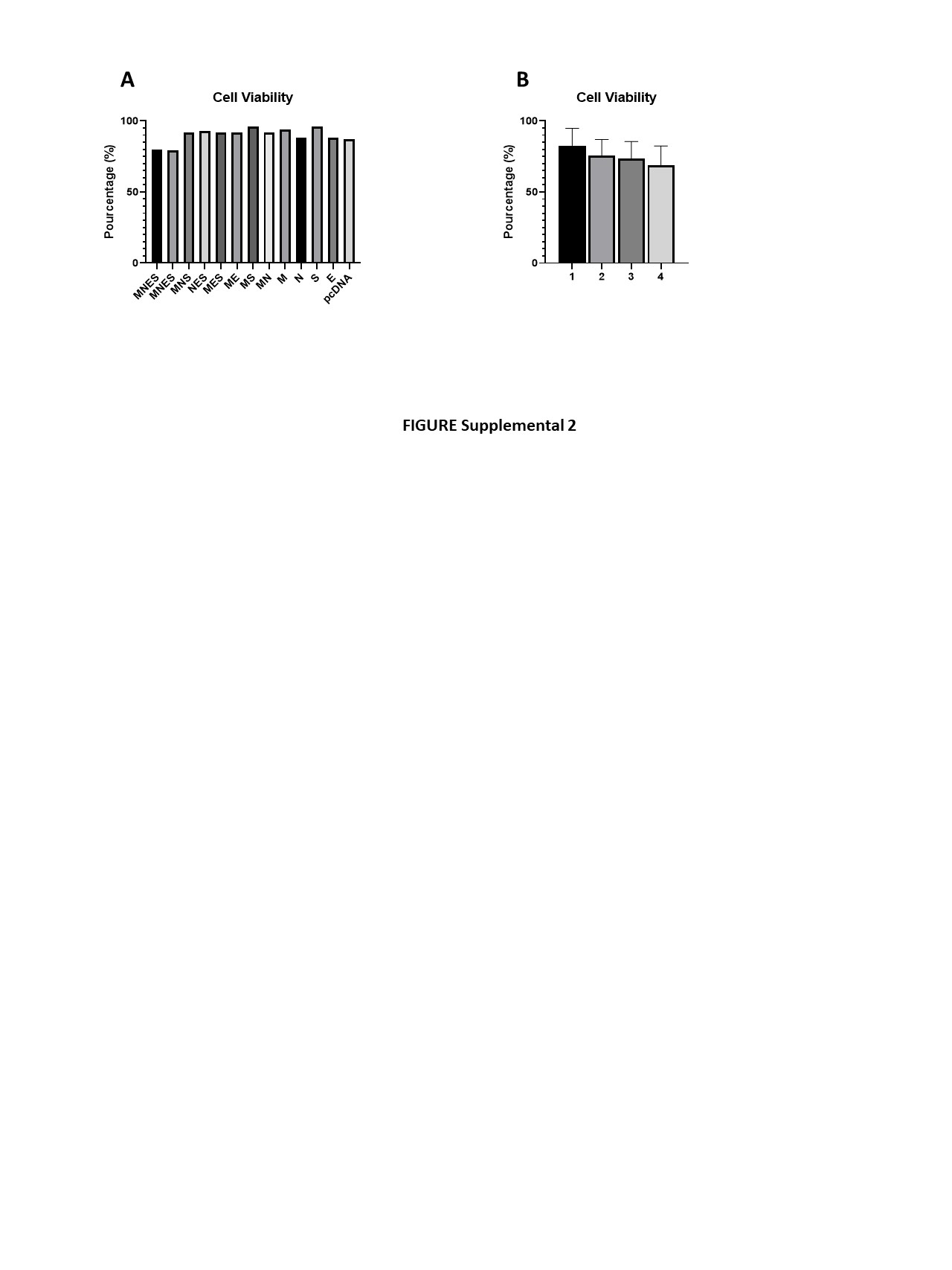
**

**C**

**
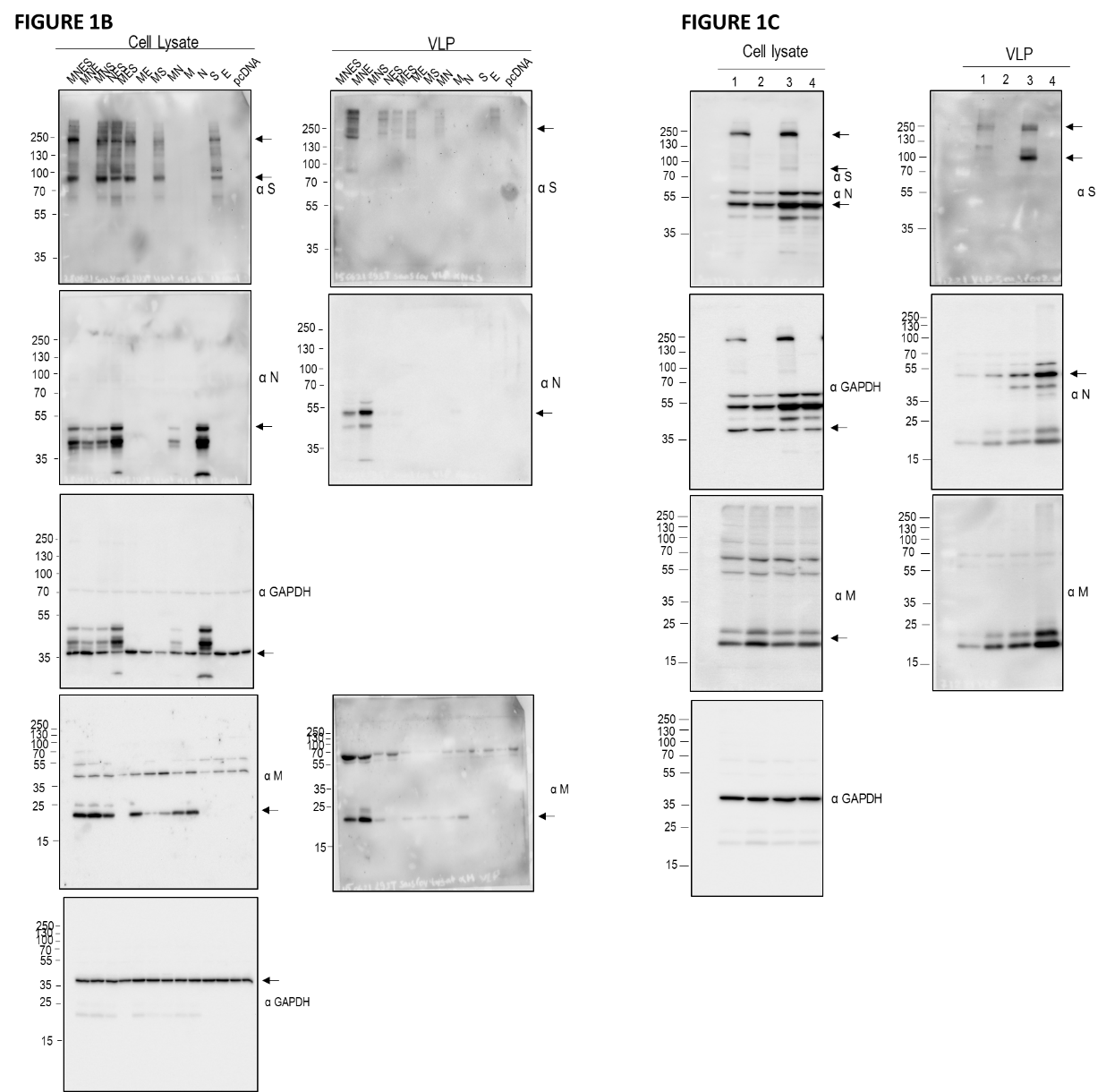
**

**D**

**
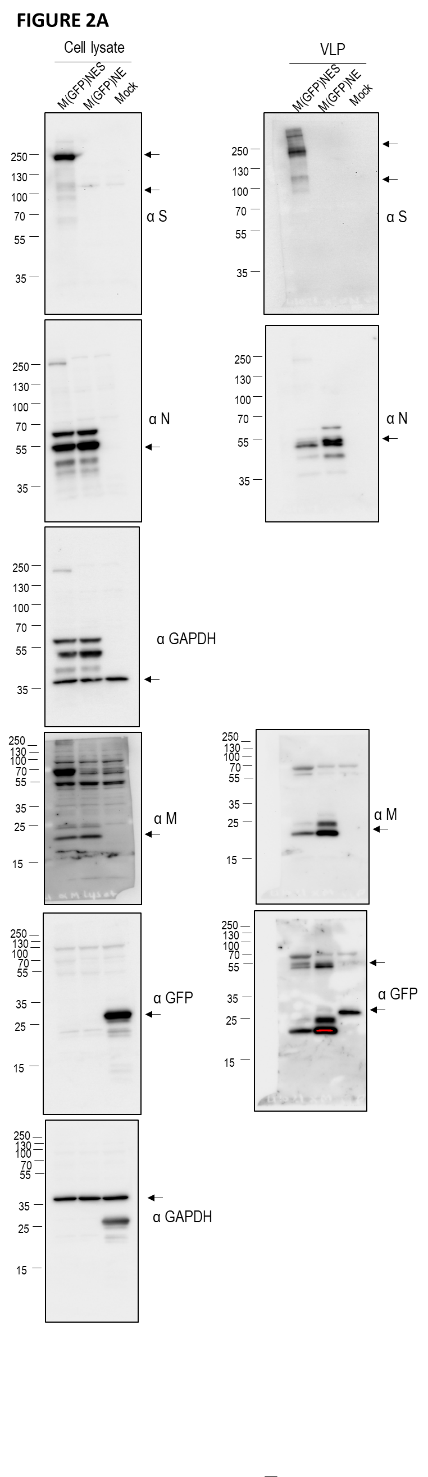
**

**E**

**
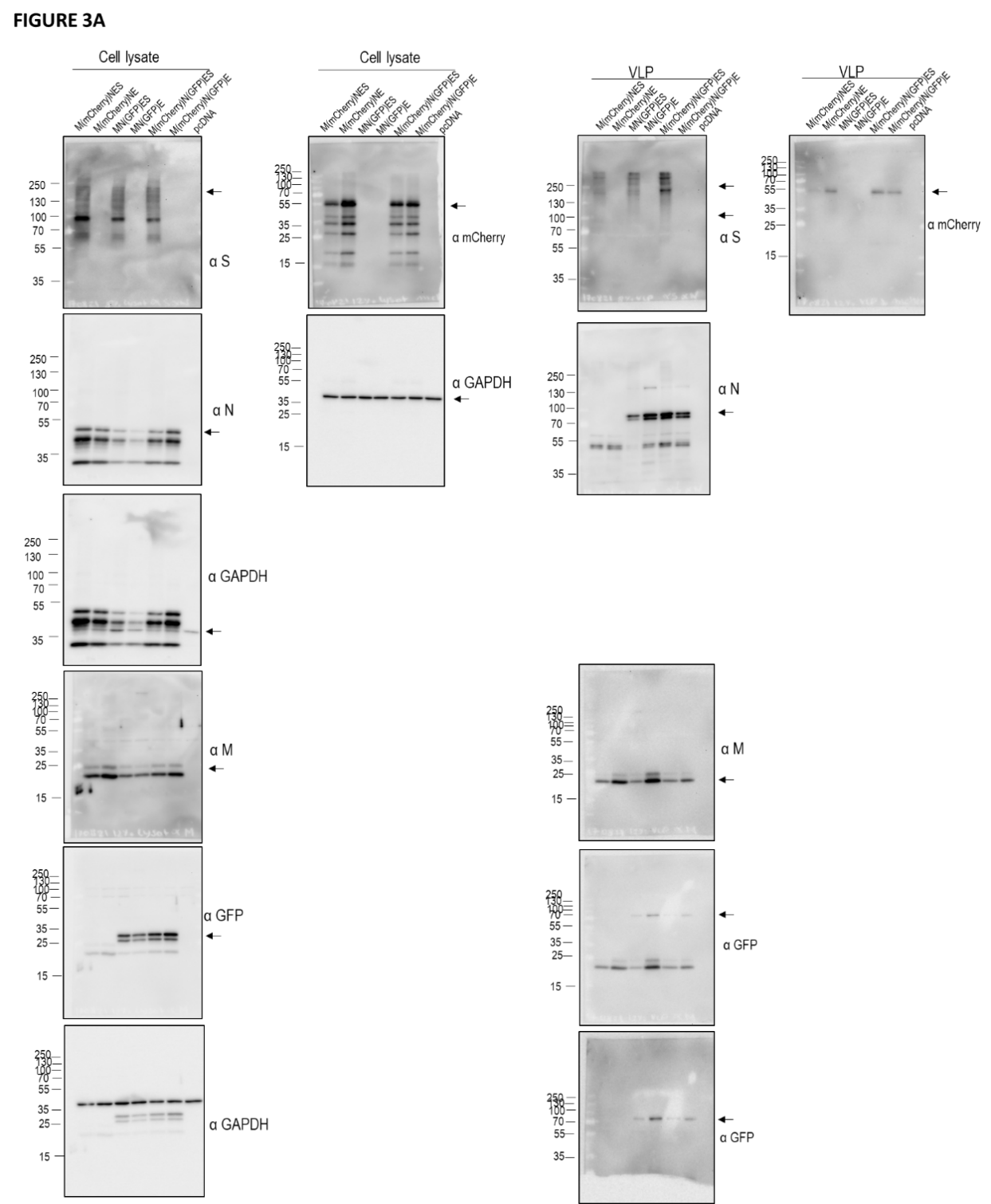
**

**Supplementary Figure 2:** **Cell viability and Western Blots of transfected HEK293T cells when producing VLPs for different experimental conditions.** (A) Cell viability of assays based on constant ratio corresponding to Figure 1B. (B) Cell viability of assays based on mimicking WT SARS-CoV-2 mRNA ratio corresponding to Figure 1C. (C) Complete Western Blots for Figure 1B and 1C for SARS-CoV-2 S, M and N in cell lysates and VLP, as indicated. (D, E) Complete Western Blots for Figure 2A and Figure 3A, respectively, for SARS-CoV-2 S, M, N and also GFP or mCherry content in cell lysates and in fluorescent VLP, as indicated. The cellular GAPDH as a loading control for cell lysates.


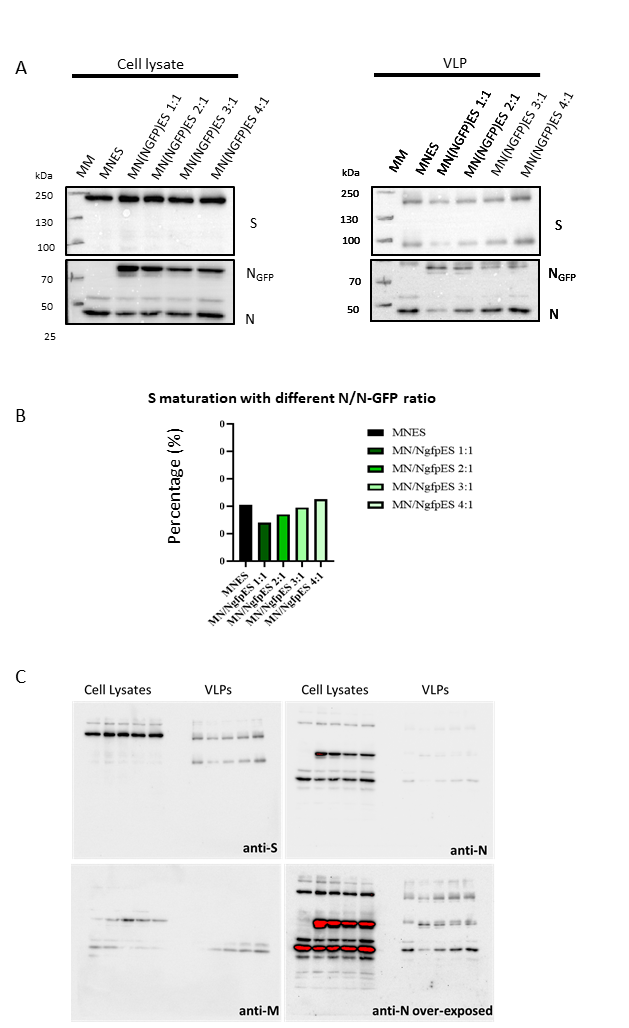


**New Supplementary Figure 3:** **S maturation in MN(Ngfp)ES VLPs in function of different N/Ngfp ratio.** (A) Western Blots of transfected HEK293T cells when producing N-gfp VLPs with different ratio of N/N-gfp in MNES VLP conditions. Western Blots show SARS-CoV-2 S, N and Ngfp in cell lysates or in VLP. Immature unprocessed Spike appears at 180 kDa and the processed mature Spike below at 80kDa. The cellular GAPDH as a loading control for cell lysates. (B) Quantification of the mature S ( %) in function of the different N/Ngfp ratio in VLPs. (C) Uncropped western blots anti-S, anti-M and anti-N for cell lysates and VLPs corresponding to panel (A).

**
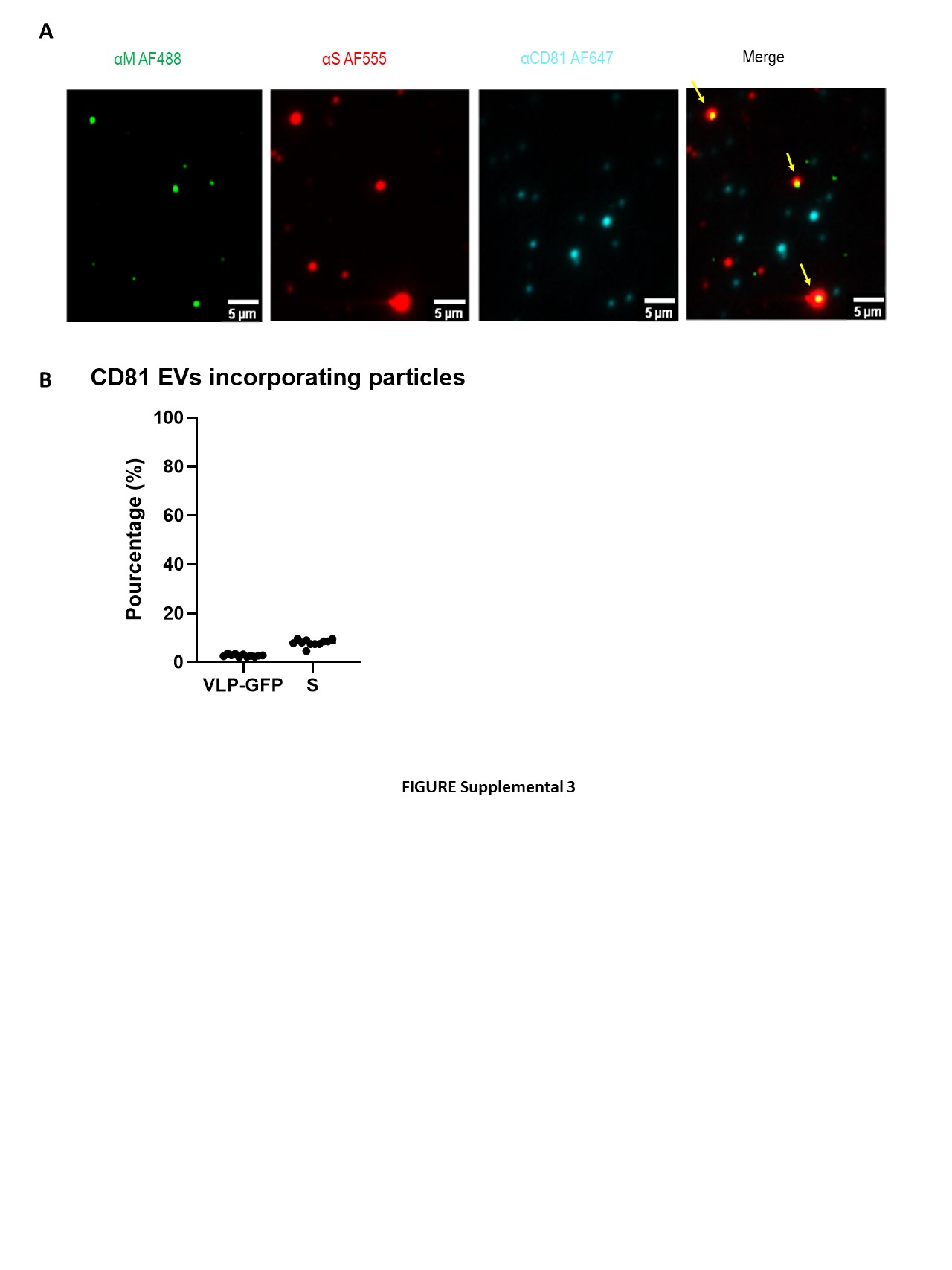
Supplementary Figure 4**: **CD81(+) EVs and SARS-CoV-2 M(GFP)NES VLPs are distinct entities as revealed by immuno-spotting coupled to TIRF-M**. (A) Images showing incorporation of the M(GFP)NES VLP on CD81-exosomes labelled with M(GFP), with a neutralizing antibody anti-S coupled with secondary AF555 antibody and an antibody anti-CD81 coupled with secondary AF647using immuno-spotting coupled to TIRF-M, showing that M(GFP)NES can contain CD81 but CD81 exosomes are not containing M(GFP) or S. Scale bar is 5µm. (B) Percentage of incorporation of M(GFP) on CD81(+) EVs showing that CD81(+) exosomes are not containing M(GFP) or S.

**
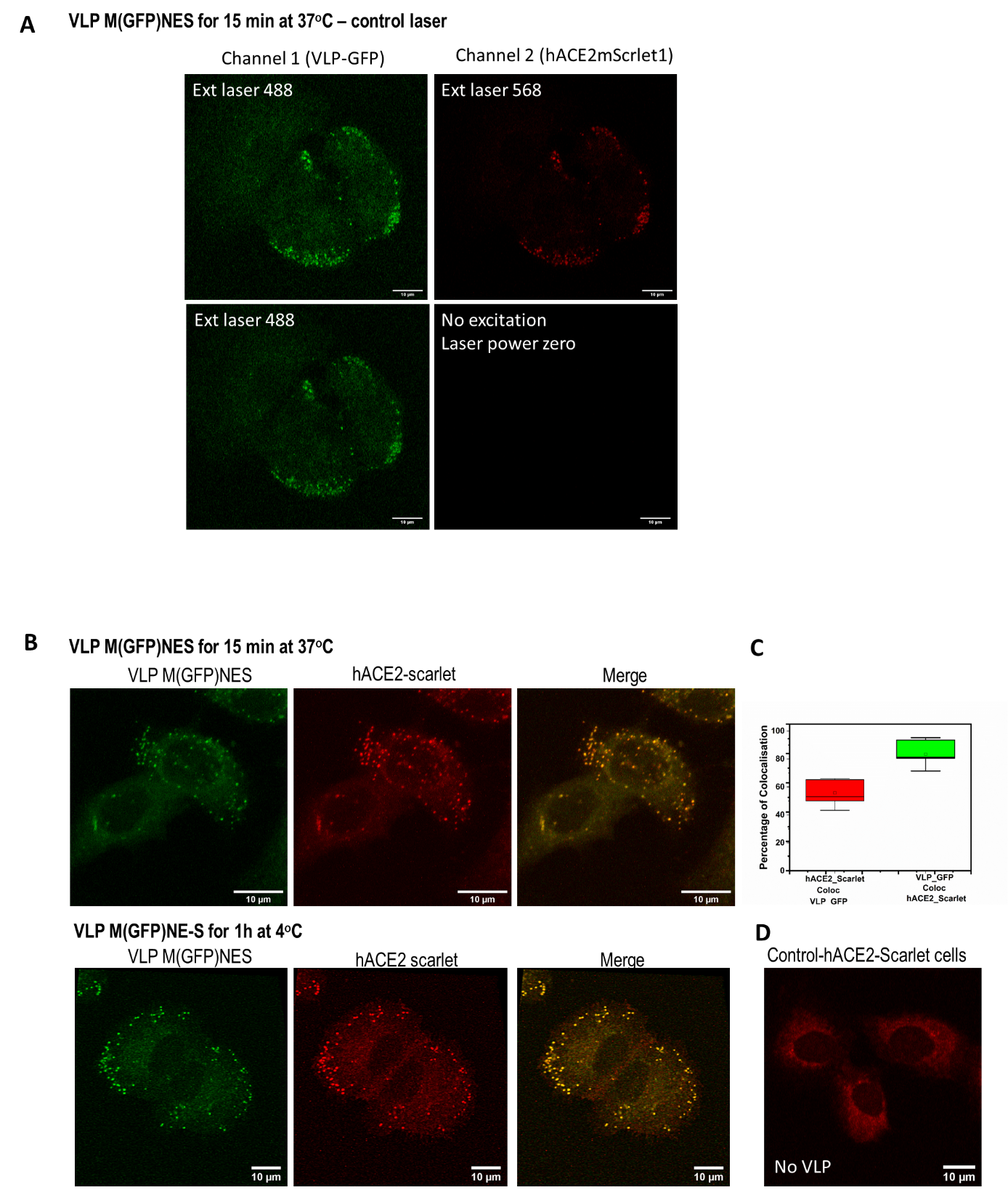
**

**New Supplementary Figure 5**:  **Imaging of VLP(M-GFP) in A549-hACE2mScarlet1 cells using confocal laser microscopy.** (A) Confocal images of VLP(GFP) on A549hACE2mScarlet1 cells showing that the GFP fluorescence (488nm excitation) is not emitting into the mScarlet1 channel (561nm excitation). Without excitation of mScarlet1 the “red color” dots do not appear on the image. (B) Confocal images of M(GFP)NES VLPs (in green) and A549-hACE2-mScarlet1 (in red) at 37°C (upper panel) and at 4°C (lower panels) showing internalization of VLPs at 37°C. (C) Percentage of colocalization of hACE2-mScarlet1 in M(GFP)NES VLPs (in red) and of M(GFP)NES VLPs in hACE2-mScarlet1 (in green) showing that most of the labelled VLPs are surrounded by hACE2-mScarlet1 receptor. (D) Image of a cell expressing hACE2-mScarlet1 without VLPs (control – No VLP).
